# Effects of social distancing and isolation on epidemic spreading modeled via dynamical density functional theory

**DOI:** 10.1038/s41467-020-19024-0

**Published:** 2020-11-04

**Authors:** Michael te Vrugt, Jens Bickmann, Raphael Wittkowski

**Affiliations:** Institut für Theoretische Physik, Center for Soft Nanoscience, Westfälische Wilhelms-Universität Münster, D-48149 Münster, Germany

**Keywords:** Diseases, Physics

## Abstract

For preventing the spread of epidemics such as the coronavirus disease COVID-19, social distancing and the isolation of infected persons are crucial. However, existing reaction-diffusion equations for epidemic spreading are incapable of describing these effects. In this work, we present an extended model for disease spread based on combining a susceptible-infected-recovered model with a dynamical density functional theory where social distancing and isolation of infected persons are explicitly taken into account. We show that the model exhibits interesting transient phase separation associated with a reduction of the number of infections, and allows for new insights into the control of pandemics.

## Abstract (web summary)

Existing models describing epidemic spreading need an update to capture effects of social distancing and isolation. An extended model is proposed by te Vrugt et al. by drawing an analogy between persons and diffusing particles with repulsive interactions that correspond to social distancing.

## Introduction

Controlling the spread of infectious diseases, such as the plague^1^ or the Spanish flu^2^, has been an important topic throughout human history^3^. Currently, it is of particular interest owing to the worldwide outbreak of the coronavirus disease 2019 (COVID-19) induced by the novel coronavirus SARS-CoV-2^4–7^. The spread of this disease is difficult to control, as the majority of infections are not detected^8^. Owing to the fact that a vaccine does not currently exist, the response to the pandemic has focused on nonpharmaceutical interventions^9^, involving the general reduction of social interactions, and in particular the isolation of persons with actual or suspected infection. For political decisions on such measures, it is important to have available a way for predicting their effects.

Mathematical models for disease spreading exist in a variety of forms. Simple compartmental models, such as the widely used susceptible-infected-recovered (SIR) model^10^, can take into account social restrictions in an effective way by changing the transmission rate, but cannot model them explicitly. Individual-based models^11^, on the other hand, are computationally very expensive in practical applications and provide little room for analytical insights. In physics, bridging the gap between large-scale and detailed descriptions is possible by using coarse-grained field theories such as dynamical density functional theory (DDFT)^12,13^, which are more efficient than particle-based simulations while still having a clear connection to the microscopic dynamics. This indicates that similar approaches will also be valuable for epidemiology.

In this article, we present a DDFT for epidemic spreading that allows to model the effects of social distancing and isolation on infection numbers. Our model is based on combining the general idea of a reaction–diffusion DDFT from soft matter physics with the SIR model from theoretical biology. The phase diagram predicted by our model shows that, at parameter values corresponding to certain strengths and ratios of social distancing and self-isolation, the system undergoes a phase transition to a state where the spread of the pandemic is suppressed. For all regions of the phase diagram, the predicted curves have the shape that is observed in real pandemics. Numerically, the inhibition of epidemic spreading is found to be associated with transient phase separation, where infected persons accumulate at certain spots. Another mechanism decreasing infection numbers is a reduction of the density. Our results are of high interest for the control of pandemics, as the effects of social distancing and the properties of the disease can be studied separately. Moreover, the observed phase separation effects can also be expected to occur in crowded (bio-)chemical systems, which are governed by similar equations.

This article is structured as follows: In the results section, we first introduce the SIR-DDFT model. Second, analytical results concerning disease outbreaks are discussed. Third, we present numerical results showing that social interactions can reduce infection numbers in our model. Fourth, the associated spatiotemporal dynamics is explained. We summarize our findings in the discussion section. In the methods section, we present details on the construction of the model, linear stability, front propagation, the basic reproduction number, and the applied numerical methods.

## Results

### SIR-DDFT model

A quantitative understanding of disease spreading can be gained from mathematical models^14–17^. In compartmental models^10^, the population is divided into various compartments that often represent different health conditions, such as susceptible or infected. The dynamical equations then describe transitions between these compartments, such as infections or recovery. In contrast, individual-based models use a bottom–up approach by considering the behavior of individual agents, which may have different locations, physical properties, and social behavior^11^. Although being more detailed than compartmental models, they are computationally much more costly.

The analog of an individual-based model in soft matter physics is a particle-based simulation, which is used for a microscopic description of a complex fluid. An alternative method used in this area are coarse-grained field theories, which describe in a simplified way the microscopic dynamics they are derived from on a larger scale. A paradigmatic example of such a theory is DDFT^18,19^. The results obtained from coarse-grained theories are often in excellent agreement with particle-based simulations and have a clear connection to the microscopic description, while providing additional analytical insights^20^. At the same time, coarse-grained theories are much easier to solve numerically. This motivates the application of such field theories, and in particular of DDFT, to the case of disease spreading.

A well-known compartmental theory for epidemic dynamics is the SIR model^10^

1 $$\dot{\bar{S}}=-{c}_{{\rm{eff}}}\bar{S}\bar{I},$$

2 $$\dot{\bar{I}}={c}_{{\rm{eff}}}\bar{S}\bar{I}-w\bar{I},$$

3 $$\dot{\bar{R}}=w\bar{I},$$

which has already been applied to the current coronavirus outbreak^21,22^. It is a reaction model that describes the number of susceptible $\bar{S}$, infected $\bar{I}$, and recovered $\bar{R}$ individuals as a function of time *t*. Susceptible individuals get the disease when meeting infected individuals at a rate *c*_eff_ (“transmission rate”, also known as “effective contact rate”^23^). For ease of notation, we have absorbed the total population size *N* into the transmission rate. Infected persons recover from the disease at a rate *w*. When persons have recovered, they are immune to the disease. (We use overbars to distinguish population numbers from population densities: $\bar{S}$ is the total number of susceptibles, while *S* is the number of susceptibles per unit area.)

A drawback of this model is that it describes spatially homogeneous dynamics, i.e., it does not take into account the fact that healthy and infected persons are not distributed homogeneously in space, even though this fact can have significant influence on a pandemic^24,25^. To allow for spatial dynamics, disease-spreading theories such as the SIR model have been extended to reaction–diffusion equations^26–32^. For this purpose, a term *D*_*ϕ*_∇^2^*ϕ*(**r**, *t*) with diffusion constant *D*_*ϕ*_ and spatial variable **r** is added on the right-hand side of the dynamical equation for *ϕ* = *S*, *I*, *R* from the SIR model (Eqs. (1–3)) with $\bar{S}$, $\bar{I}$, and $\bar{R}$ replaced by the corresponding densities *S*, *I*, and *R* and *c*_eff_ replaced by a parameter *c* that has dimensions of area/time. No change is required for the parameter *w*. Using a reaction–diffusion equation to model disease spreading corresponds to assuming local transmissions^33^. A phenomenological extension incorporates the avoidance of infected persons by susceptible persons using cross-diffusion^34^. This, however, does not incorporate general physical distancing also between healthy persons (which is required if infected persons cannot generally be identified as such).

A drawback of reaction–diffusion equations is that they—being based on the standard diffusion equation—do not take into account particle interactions other than the reactions. This issue arises, e.g., in chemical reactions in crowded environments such as inside a cell. If the number of reactions is limited by the rate of encounters, it will be reduced by crowding^35^. To get an improved model, one can make use of the fact that the diffusion equation is a special case of DDFT. In this theory, the time evolution of a density field *ρ*(**r**, *t*) is given by

4 $${\partial }_{t}\rho =\Gamma \nabla \cdot \left(\rho \nabla \frac{\delta F}{\delta \rho }\right)$$

with a mobility *Γ* and a free-energy functional *F*. Note that we have written Eq. (4) without noise terms, which implies that *ρ*(**r**, *t*) denotes an ensemble average^36^. The free energy is given by

5 $$F={F}_{{\rm{id}}}+{F}_{{\rm{exc}}}+{F}_{{\rm{ext}}}.$$

Its first contribution is the ideal gas free energy

6 $${F}_{{\rm{id}}}={\beta }^{-1}\mathop{\int}\nolimits_{}^{}{{\rm{d}}}^{d}r\rho ({\bf{r}},t)(\mathrm{ln}\,(\rho ({\bf{r}},t){\Lambda }^{d})-1),$$

corresponding to a system of noninteracting particles with the rescaled inverse temperature *β*, number of spatial dimensions *d*, and thermal de Broglie wavelength *Λ*. If this is the only contribution, Eq. (4) reduces to the standard diffusion equation with *D* = *Γβ*^−1^. The second contribution is the excess free energy *F*_exc_, which takes the effect of particle interactions into account. It is typically not known exactly and has to be approximated. The third contribution *F*_ext_ incorporates the effect of an external potential *U*_ext_(**r**, *t*). DDFT can be extended to mixtures^37,38^, which makes it applicable to chemical reactions. Although DDFT is not an exact theory (it is based on the assumption that the density is the only slow variable in the system^18,19^), it is nevertheless a significant improvement compared to the standard diffusion equation as it allows to incorporate the effects of particle interactions and generally shows excellent agreement with microscopic simulations. In particular, it allows to incorporate the effects of particle interactions such as crowding in reaction–diffusion equations. This is done by replacing the diffusion term *D*∇^2^*ϕ*(**r**, *t*) in the standard reaction–diffusion model with the right-hand side of the DDFT equation (4)^39–42^. Thus, given that both static density functional theory (DFT)^43^ and dynamical models for interacting agents^44–51^ have previously been used to describe social systems, DDFT is a very promising approach for the development of extended models for epidemic spreading. In particular, the successes of DDFT in other biological contexts such as cancer growth^52^, protein adsorption^53^, ecology^54^, or active matter^55–61^ suggest that it can be an extremely valuable tool also in the present context.

We use the idea of a reaction–diffusion DDFT to extend the SIR model given by Eqs. (1–3) to a (probably spatially inhomogeneous) system of interacting persons, which compared to existing methods allows the incorporation of social interactions and in particular of social distancing. Persons are modeled as diffusing particles that can be susceptible to, infected with, or recovered from a certain disease. Social distancing and self-isolation are incorporated as repulsive interactions. The dynamics of the interacting particles can then be described by DDFT, while reaction terms account for disease transmission and recovery. DDFT describes the diffusive relaxation of an interacting system and is thus appropriate if we make the plausible approximation that the underlying diffusion behavior of persons is Markovian^62^ and ergodic^63^. Using the Mori–Zwanzig formalism^64–66^, one can connect the DDFT model and its coefficients to the dynamics of the individual persons^18,19^. The extended model reads

7 $${\partial }_{t}S={\Gamma }_{S}\nabla \cdot \left(S\nabla \frac{\delta F}{\delta S}\right)-cSI,$$

8 $${\partial }_{t}I={\Gamma }_{I}\nabla \cdot \left(I\nabla \frac{\delta F}{\delta I}\right)+cSI-wI-mI,$$

9 $${\partial }_{t}R={\Gamma }_{R}\nabla \cdot \left(R\nabla \frac{\delta F}{\delta R}\right)+wI.$$

Note that we allow for different mobilities *Γ*_*S*_, *Γ*_*I*_, and *Γ*_*R*_ for the different fields *S*, *I*, and *R*. For generality, we have added a term –*mI* on the right-hand side of Eq. (8) to allow for death of infected persons, which occurs at a rate *m* (cf. SIRD model^67,68^). Details on the microscopic construction of the extended model can be found in the first part of the methods section. Since we are mainly interested in how fast the infection spreads, we set *m* = 0 in the following. In this case, as the total number of persons is constant, one can easily show that

10 $${\bf{J}}=-{\Gamma }_{S}S\nabla \frac{\delta F}{\delta S}-{\Gamma }_{I}I\nabla \frac{\delta F}{\delta I}-{\Gamma }_{R}R\nabla \frac{\delta F}{\delta R}$$

is a conserved current. The ideal gas term *F*_id_ in the free energy corresponds to a system of noninteracting persons and ensures that standard reaction–diffusion models for disease spreading^28^ arise as a limiting case. The temperature measures the intensity of motion of the persons. A normal social life corresponds to an average temperature, whereas the restrictions associated with a pandemic will lead to a lower temperature. Moreover, the temperature can be position-dependent if the epidemic is dealt with differently in different places. The excess free energy *F*_exc_ describes interactions. This is crucial here as it allows to model effects of social distancing and self-isolation via a repulsive potential between the different persons. Social distancing is a repulsion between healthy persons, while self-isolation corresponds to a stronger repulsive potential between infected persons and other persons. Thus, we set

11 $${F}_{{\rm{exc}}}={F}_{{\rm{sd}}}+{F}_{{\rm{si}}}$$

with *F*_sd_ describing social distancing and *F*_si_ self-isolation. Note that effects of such a repulsive interaction are not necessarily covered by a general reduction of the diffusivity in existing reaction–diffusion models. For example, if people practice social distancing, they will keep a certain distance in places such as supermarkets, where persons accumulate even during a pandemic, or if people live in crowded environments, as was the case on the ship Diamond Princess^69^. In our model, in the case of two particles approaching each other, which even at lower temperatures still happens, repulsive interactions will reduce the probability of a collision and thus of an infection. Existing models can only incorporate this in an effective way as a reduction of the transmission rate *c*_eff_, which implies, however, that properties of the disease (How infectious is it?) and measures implemented against it (Do people stay away from each other?) cannot be modeled independently. Furthermore, interactions allow for the emergence of spatiotemporal patterns. The final contribution is the external potential *U*_ext_. In general, it allows to incorporate effects of confinement into DDFT. Here, it corresponds to things such as externally imposed restrictions of movement. Travel bans or the isolation of a region with high rates of infection enter the model as potential wells.

The advantage of our model compared to the standard SIR theory is that it allows—in a way that is computationally much less expensive than microscopic simulations, as the computational cost of a DDFT calculation is independent of the number of persons for a fixed system size and resolution^70^—to study the way in which different actions affect how the disease spreads. For example, people staying at home corresponds to reducing the temperature, social distancing corresponds to repulsive interaction potentials, and mass events correspond to attractive potentials.

Specifically, we assume that both types of interactions can be modeled via Gaussian pair potentials, depending on the parameters *C*_sd_ and *C*_si_ determining the strength and *σ*_sd_ and *σ*_si_ determining the range of the interactions (parameters with subscript sd account for social distancing, parameters with subscript si for self-isolation). This corresponds to describing the society as a Gaussian core model (GCM) fluid^37,71–74^. Gaussian interaction potentials are widely used to model, e.g., the behavior of polymers. They capture in an effective way the fact that polymers are penetrable objects that cannot be described as hard spheres. In DFT, GCM fluids are well described by a mean-field approximation^71,72^. For describing social interactions, soft interaction potentials are more appropriate than hard-core potentials: Persons can still get very close to each other if they practice social distancing, they are just less likely to. Different ways to practice social distancing can be accounted for by varying the form of the interaction potential.

Combining the Gaussian pair-interaction potential with a mean-field approximation^72,75^ for the excess free energy, we get the specific SIR-DDFT model

12 $$\begin{array}{ccc}{\partial }_{t}S&=&{D}_{S}{\nabla }^{2}S-{\Gamma }_{S}\nabla \cdot \left(S\nabla \left({C}_{{\rm{sd}}}{K}_{{\rm{sd}}}\star (S+R)\right.\right.\\ &&\left.\left.\, +\, {C}_{{\rm{si}}}{K}_{{\rm{si}}}\star I\right)\right)-cSI,\end{array}$$

13 $$\begin{array}{ccc}{\partial }_{t}I&=&{D}_{I}{\nabla }^{2}I-{\Gamma }_{I}\nabla \cdot \left(I\nabla ({C}_{{\rm{si}}}{K}_{{\rm{si}}}\star (S+I+R))\right)\\ &&+cSI-wI,\end{array}$$

14 $$\begin{array}{ccc}{\partial }_{t}R&=&{D}_{R}{\nabla }^{2}R-{\Gamma }_{R}\nabla \cdot \left(R\nabla \left({C}_{{\rm{sd}}}{K}_{{\rm{sd}}}\star (S+R)\right.\right.\\ &&+\left.\left.{C}_{{\rm{si}}}{K}_{{\rm{si}}}\star I\right)\right)+wI\end{array}$$

with the diffusion coefficients *D*_*ϕ*_ = *Γ*_*ϕ*_*β*^−1^ for *ϕ* = *S*, *I*, *R*, the kernels

15 $${K}_{{\rm{sd}}}({\bf{r}})=\exp (-{\sigma }_{{\rm{sd}}}{{\bf{r}}}^{2}),$$

16 $${K}_{{\rm{si}}}({\bf{r}})=\exp (-{\sigma }_{{\rm{si}}}{{\bf{r}}}^{2}),$$

and the spatial convolution ⋆. A possible generalization is discussed in Supplementary Note 1.

### Disease outbreak

We perform a linear stability analysis of this model, using a general pair potential, in order to determine whether a homogeneous state with *I* = 0, which is always a fixed point, is stable. This provides an analytical criterion for whether a disease outbreak will occur. The full calculation is given in the second part of the methods section. In the simple SIR model, a fixed point with $\bar{S}={\bar{S}}_{0}$, where ${\bar{S}}_{0}$ is a constant, is unstable when ${c}_{{\rm{eff}}}{\bar{S}}_{0}\, > \, w$^33^. Thus, the pandemic cannot break out if persons recover faster than they are able to infect others. A linear stability analysis of the full model gives the eigenvalue

17 $${\lambda }_{1}=c{S}_{\text{hom}}-w-{D}_{I}{k}^{2}$$

with the wavenumber *k* and the homogeneous reference density of susceptibles *S*_hom_, such that a homogeneous state with density *S*_hom_ is unstable for *cS*_hom_ > *w*. As reported in the literature^28^, the marginal stability hypothesis^76–80^ gives, based on this dispersion, a front propagation speed of $v=2\sqrt{{D}_{I}(c{S}_{\text{hom}}-w)}$. (This is shown explicitly in the third part of the methods section.) However, there are two additional eigenvalues *λ*_2_ and *λ*_3_ associated with instabilities owing to interactions.

The outbreak criterion obtained from Eq. (17) can be directly translated into the well-known criterion *R*_0_ > 1, where *R*_0_ is the basic reproduction number^81^ (this is shown in the fourth part of the methods section), as well as into the criterion ${c}_{{\rm{eff}}}{\bar{S}}_{0}> w$ from the SIR model (this is shown in the second part of the methods section). In general, however, the situation is more complex in the presence of spatiotemporal dynamics and interactions. To see this, note that Eq. (1) from the standard SIR model can be derived by integrating Eq. (7), which is the small-scale description, over space (a region of space with no flux of people through the boundaries). This motivates the subscript eff, which we use for the transmission rate in the standard SIR model: Actually, it is an effective transmission rate *c*_eff_, which is related to the parameter *c* by

18 $${c}_{{\rm{eff}}}(t)=c\mathop{\int}\nolimits_{}^{}{{\rm{d}}}^{d}r{e}_{S}({\bf{r}},t){e}_{I}({\bf{r}},t)$$

with the normalized distributions ${e}_{S}=S/\bar{S}$ and ${e}_{I}=I/\bar{I}$. Hence, the transmission rate *c*_eff_ observed on large scales depends on the spatial overlap of the functions *S* and *I*. If the infected persons are spatially isolated from the susceptible persons, there will be no infections even if the number of infected persons is relatively large. (In the standard SIR model, the contact rate enters *c*_eff_ as a factor^23^.) Consequently, the integral on the right-hand side of Eq. (18) can be decreased by reducing the number of contacts between susceptible and infected persons, and can thus account for the effects of social restrictions. The effects of measures such as face masks that affect *c* can be studied separately in our model, and can thus be distinguished from the effects of a change in the spatial distribution.

Equation (18) shows that there are, in principle, two mechanisms by which the repulsive interactions can lead to a reduction of the transmission rate *c*_eff_: First, the spatial overlap of *S* and *I* can be reduced by isolating the infected from the susceptible persons. Second, even if no demixing of this type occurs, people may spread over a larger distance if they repel each other. For a completely homogeneous distribution, *c*_eff_ is just inversely proportional to the domain area, such that a reduction of *c*_eff_ is possible by increasing the area over which persons are distributed. This is, as discussed in the fourth part of the methods section, the reason why *R*_0_ is smaller in rural areas. A detailed discussion of the effective transmission rate can be found in Supplementary Note 2.

### Inhibition of epidemic

For a further analysis, we solved Eqs.  (12–14) numerically in two spatial dimensions with periodic boundary conditions. Details on the simulations can be found in the fifth part of the methods section. The relevant control parameters are *C*_sd_ and *C*_si_, which determine the strength of social interactions that are the new aspect of our model. We assume these parameters to be ≤0, which corresponds to repulsive interactions. Moreover, we assume **r** and *t* to be dimensionless, such that all model parameters can be dimensionless too. As is common, we present our results in terms of the fraction of the total population that is susceptible (${\bar{S}}_{{\rm{n}}}=\bar{S}/N$), infected (${\bar{I}}_{{\rm{n}}}=\bar{I}/N$), or recovered (${\bar{R}}_{{\rm{n}}}=\bar{R}/N$). In Supplementary Note 3, we provide results of simulations in one spatial dimension and for a smaller domain in order to assess the way in which the number of dimensions and the domain size affect the results.

Measures implemented against a pandemic will typically have two aims: enlargement of ${\bar{S}}_{\infty ,{\rm{n}}}={\mathrm{lim}\,}_{t\to \infty }{\bar{S}}_{{\rm{n}}}(t)$, which (for *R*(**r**, 0) = 0) is the fraction of persons that are not infected during the pandemic, and reduction of the maximum fraction of infected persons ${\bar{I}}_{\max ,{\rm{n}}}$ for keeping the spread within the capacities of the healthcare system. Using parameter scans, we can test whether social distancing and self-isolation can achieve those effects.

In particular, we wish to describe an outbreak within a city, i.e., a medium-size spatial region in which it can be reasonably assumed that people move primarily via diffusive random walks and not using airplanes or trains. The case of a city is of special interest, as the population density in cities is relatively large, which can lead to particularly severe outbreaks that have to be controlled on a local scale. Moreover, systematic investigations of local spreading can lead to important insights into the properties of the disease. A study with this aim was performed for the COVID-19 outbreak in the district of Heinsberg (Germany) by Streeck et al.^82^. Individual-based models, which allow to incorporate contact networks, are a typical approach for modeling cities^83^. Transportation networks can also be included in DDFT, where streets can be represented by confining potentials (that would represent narrow channels in usual fluid mechanics). Here, we assume a spatially homogeneous city for simplicity.

**Fig. 1:**
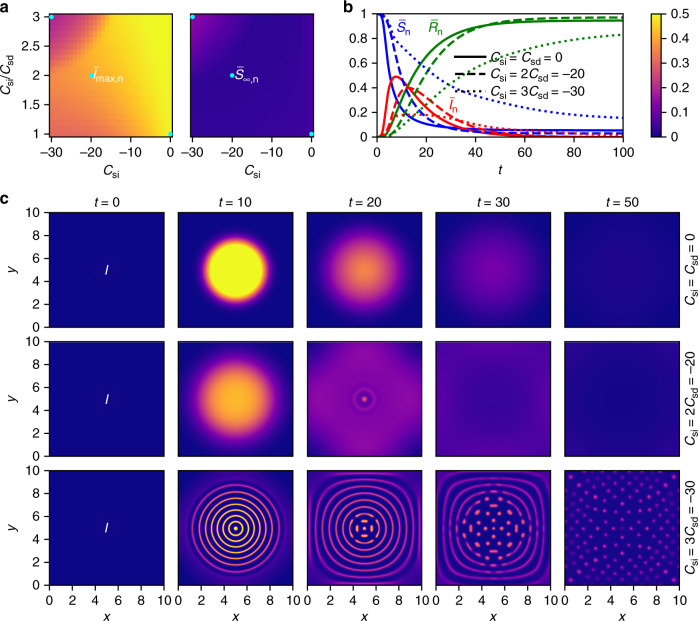
Phase diagrams and time evolutions for the SIR-DDFT model. **a** The shown phase diagrams reveal the dependence of the maximal fraction of infected persons ${\bar{I}}_{\max ,{\rm{n}}}$ and the final fraction of susceptible persons ${\bar{S}}_{\infty ,{\rm{n}}}$ on the strength of self-isolation *C*_si_ and social distancing *C*_sd_. A phase boundary is clearly visible. Blue points correspond to the time evolutions presented in the following subfigures. **b** Time evolutions of the fractions of susceptible (${\bar{S}}_{{\rm{n}}}$), infected (${\bar{I}}_{{\rm{n}}}$), and recovered (${\bar{R}}_{{\rm{n}}}$) persons are shown for no interactions (*C*_si_ = *C*_sd_  = 0), moderate interactions (*C*_si_ = 2*C*_sd_ = −20), and strong interactions (*C*_si_ = 3*C*_sd_ = −30). It can be seen that a reduction of social contacts flattens the curve ${\bar{I}}_{{\rm{n}}}(t)$. **c** The density of infected persons *I*(*x*, *y*, *t*) is shown for the same three interaction strengths at different times *t*. For the strongest interactions, phase separation (first into rings, then into single spots) is observed. The color bar applies to Figs. 1a and 1c.

As a first scenario, we consider an initial Gaussian accumulation of people in the middle of the domain. This allows us to model the infection dynamics after a super-spreading event, for which the outbreak in Heinsberg that took place after a traditional carnival festivity (Kappensitzung)^82^ is a good example. As can be seen from the phase diagrams for the SIR-DDFT model shown in Fig. 1a, there is a clear phase boundary between the upper left corner, where low values of ${\bar{I}}_{\max ,{\rm{n}}}$ and high values of ${\bar{S}}_{\infty ,{\rm{n}}}$ show that the spread of the disease has been significantly reduced, and the rest of the phase diagrams. This is, as shown in the fourth part of the results section, a consequence of phase separation. In addition, a smaller reduction of ${\bar{I}}_{\max ,{\rm{n}}}$ is observed in the bottom left corner of the phase diagram, where ∣*C*_si_∣ and ∣*C*_sd_∣ are large and have similar values. There, the diffusive spread after a Gaussian initial distribution, which is altered by the presence of interactions (the distribution is then broader at early times), is a relevant mechanism. As all simulations were performed with parameters of *c* and *w* that allow for a disease outbreak by the criterion presented in the second part of the results section already for small values of *S*_hom_, our results show that a reduction of social interactions can significantly inhibit epidemic spreading, and that the SIR-DDFT model is capable of demonstrating these effects.

 Figure 1b shows the time evolutions of the fractions ${\bar{S}}_{{\rm{n}}}(t)$, ${\bar{I}}_{{\rm{n}}}(t)$, and ${\bar{R}}_{{\rm{n}}}(t)$ of susceptible, infected, and recovered persons, respectively, for the cases without interactions (usual SIR model with diffusion) and with interactions (our model). If no interactions are present (i.e., *C*_si_ = *C*_sd_ = 0), ${\bar{I}}_{{\rm{n}}}(t)$ reaches a maximum value of about 0.49 and the pandemic is over at time *t* ≈ 58 (where the end of the pandemic is the point in time where ${\bar{I}}_{{\rm{n}}}\, <\, 0.01$ for all subsequent times). In the case with interactions (we choose *C*_si_ = 3*C*_sd_ = −30, i.e., parameter values inside the social isolation phase), the maximum is significantly reduced to a value of about 0.18. The value of ${\bar{R}}_{{\rm{n}}}(t)$ at the end of the simulation, which measures the fraction of persons that have been infected during the pandemic, decreases from about 0.95 to about 0.85. Moreover, it takes significantly longer (until time *t* ≈ 106) for the pandemic to end. This demonstrates that social distancing and self-isolation have the effects they are supposed to have, i.e., to flatten the curve ${\bar{I}}_{{\rm{n}}}(t)$ in such a way that the healthcare system is able to take care of all cases. Intermediate results are found for *C*_si_ = 2*C*_sd_ = −20, where social restrictions reduce the maximum to about 0.4 (the final fraction of recovered persons actually increases to about 0.97) and the end is reached at *t* ≈ 64. Notably, all curves were obtained with the same values of *c* and *w*, i.e., the properties of the disease are identical. Hence, the observed effect is solely a consequence of social interactions. In the usual SIR model, in contrast, these would be accounted for by modifying *c*_eff_, such that they could not be studied separately. The theoretical predictions for the effects of social restrictions on the course of ${\bar{I}}_{{\rm{n}}}(t)$ (sharp rise, followed by a bend and a flat curve) are in good qualitative agreement with recent data from China^84,85^, where strict regulations were implemented to control the COVID-19 spread^86^.

Interestingly, it turns out that a flattened curve is not always completely beneficial. For intermediate interaction strengths (*C*_si_ = 2*C*_sd_ = −20), the interactions lead to a smaller value of ${\bar{I}}_{\max ,{\rm{n}}}$, but also to a slightly larger overall fraction of infected persons (smaller ${\bar{S}}_{\infty ,{\rm{n}}}$). This can be explained by the modification of the time-dependent effective transmission rate *c*_eff_ in the presence of spatiotemporal dynamics. During the initial spreading phase, *c*_eff_ is lower than in the noninteracting case. Consequently, ${\bar{I}}_{\max ,{\rm{n}}}$ is decreased. At later times, where a larger fraction of infected persons is left in the interacting case, the transmission rate is slightly larger, leading to an increased overall fraction of infected persons. For *C*_si_ = 3*C*_sd_ = −30, the effects of the interactions are strong enough to keep the transmission rate below that of the noninteracting case also at later times. A full discussion of the development of *c*_eff_ for the time evolutions presented in Fig. 1 can be found in Supplementary Note 2.

**Fig. 2:**
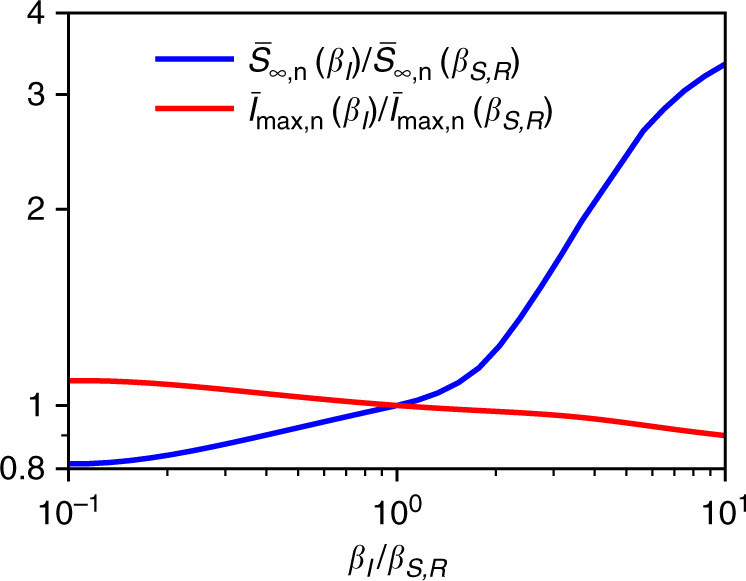
Dependence of infection numbers on the amount of motion of infected persons. The maximal fraction of infected persons ${\bar{I}}_{\max ,{\rm{n}}}$ and the final fraction of susceptible persons ${\bar{S}}_{\infty ,{\rm{n}}}$ are shown as functions of *β*_*I*_/*β*_*S*,*R*_, where *β*_*I*_ and *β*_*S*,*R*_ are the rescaled inverse temperatures corresponding to the amount of motion of the infected and healthy persons, respectively. The plot corresponds to strong interactions with *C*_si_ = 3*C*_sd_ = −30. A decrease of the temperature, i.e., a reduction of the amount of motion of the infected persons, inhibits the outbreak.

Self-isolating persons will in practice exhibit a reduced amount of motion, which in our model corresponds to a lower temperature (see the first part of the methods section for details). The effects of a reduced or increased rescaled inverse temperature *β*_*I*_ of the infected persons are shown in Fig. 2. As expected, a lower temperature reduces the spread of the disease. The effect on ${\bar{S}}_{\infty ,{\rm{n}}}$ is particularly strong.

### Spatiotemporal dynamics and phase separation

To explain the observed phenomena, it is helpful to analyze the spatial distribution of susceptible and infected persons during the pandemic. Figure 1c visualizes *I*(*x*, *y*, *t*) with *x* = (**r**)_1_ and *y* = (**r**)_2_ for times *t* =  0, 10, 20, 30, and 50 and parameter values *C*_si_ = *C*_sd_ = 0, *C*_si_ = 2*C*_sd_ = −20, and *C*_si_ = 3*C*_sd_ = −30. Without interactions (*C*_si_ = *C*_sd_ = 0), the pandemic just spreads radially and then vanishes. The infected persons form a growing disk with a high density and a sharp boundary in the middle of the domain. For moderate interactions (*C*_si_ = 2*C*_sd_ = −20), a radial spread is still observed. However, the boundary of the disk is smoothened significantly, such that the persons are distributed over a larger area and the density is lower.

The most interesting behavior can be found for large values of ∣*C*_si_∣ and *C*_si_/*C*_sd_, i.e., strong interactions: the infection spreads outwards in concentric circles that then split into separate spots. This phase separation is a consequence of the interactions. As the formation of infection rings and spots reduces the spatial overlap of the functions *S*(*x*, *y*, *t*) and *I*(*x*, *y*, *t*), i.e., the amount of contacts between susceptible and infected persons, one observes both an increase of ${\bar{S}}_{\infty ,{\rm{n}}}$ and a decrease of ${\bar{I}}_{\max ,{\rm{n}}}$, as well as a longer duration of the pandemic. This is relevant in the top left corner of the phase diagrams in Fig. 1a. Physically, the separation into small, evenly distributed infection spots is a reasonable description of infected persons that self-isolate by going home and then staying there.

The demixing transition is an interesting type of transient phase behavior in its own right. Recall that we have motivated the SIR-DDFT model based on theories for chemical reactions of interacting diffusing particles. It is thus very likely that effects similar to the ones observed here can be found in chemistry. In this case, they would imply that particle interactions can significantly affect the amount of a certain substance that is produced within a chemical reaction, and that such reactions are accompanied by new types of (transient) pattern formation. An interesting example is a system of polymers undergoing chemical reactions. If interpreted in this way, the results presented in the bottom row of Fig. 1c correspond to temporary demixing of a complex fluid. For two-dimensional systems as considered here, the minority phase can in general arrange into separated islands or into a connected network (percolation)^87^. In the present case, one can distinguish between rings and spots, with the former developing into the latter over the course of time. See refs. ^37,71–74^ for a general discussion of the phase behavior of GCM fluids.

The observation that the disease initially spreads with a circular wavefront, made for simulations without and with phase separation, is in agreement with predictions from simple reaction–diffusion SIR models^33^. These have been found to give reasonable results for, e.g., the spread of the Black Death in Europe or of Rabies in England^88^. Radial spreading is even observed in modern traffic networks on global scales, provided these are described in terms of effective distance^89^ (see the first part of the methods section). Moreover, spatial pattern formation is known in other contexts from reaction–diffusion SIR models^90^, including variants where cross-diffusion is used to describe susceptibles avoiding infected persons^34^. Empirically, hotspots (caused by other mechanisms) were observed, e.g., for the spread of HIV in South Africa^91^ or Dengue in Thailand^92^. Moreover, it was found that spatial pattern formation can facilitate the control of infectious diseases such as Rabies^93^. This agreement indicates that the predictions of our model are correct. However, the SIR-DDFT model is more general and incorporates interaction effects not present in simple diffusion models.

**Fig. 3:**
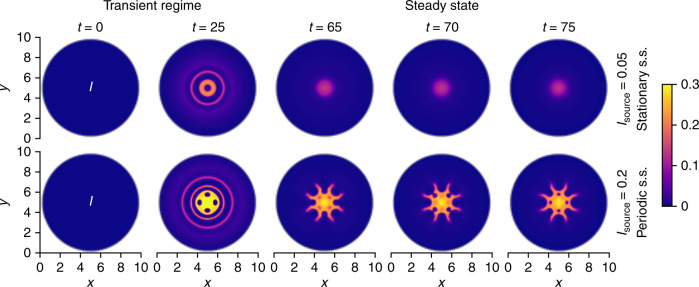
Time evolution of the density of infected persons ***I***(***x***, ***y***, ***t***) for a local source of infected persons (airport). The time evolution is shown for strong interactions with *C*_si_ = 3*C*_sd_ = −30 and different influx strengths *I*_source_. Initially, one observes radial spreading (transient regime). Later, a steady state is reached, which is stationary for a small influx. A larger influx leads to a periodic regime (*I*(*x*, *y*, *t*) = *I*(*x*, *y*, *t* + *nτ*) with $n\in {\mathbb{N}}$ and period duration *τ* ≈ 16), involving the formation of a complex oscillating structure.

As a second scenario, we consider a city with an airport, which corresponds to a local source of infected persons. We use a spherical domain and employ Dirichlet boundary conditions (see the fifth part of the methods section for details). Overpopulation is avoided by an outflux at the boundaries. The results are shown in Fig. 3. First, one again observes radial spreading in the form of infection rings (transient regime). For later times, a steady state is reached as a consequence of the fact that the number of remaining susceptibles is very low. The steady state is stationary when the influx is weak. If the influx is stronger (which is less realistic for disease spreading, but of interest for chemical applications), one observes the formation of a complex oscillating pattern as steady state.

## Discussion

We have presented a DDFT-based extension of the SIR model for epidemic spreading. It describes persons as diffusing particles with repulsive interactions that correspond to social distancing and self-isolation. The resulting theory provides more detailed insights into the spread of diseases than simpler compartmental models, whereas at the same time being much easier to handle than individual-based models.

Analytical investigations of the model revealed that the standard results for linear stability, basic reproduction number, and front propagation can be recovered. In addition, the SIR-DDFT model allows for more detailed microscopic insights into the effective transmission rate, showing that and how it can be modified by the amount of contacts. A decrease of the transmission rate is possible by an isolation of infected persons or by distributing the population over a larger area.

When solving the model numerically in two spatial dimensions to obtain its phase diagram, it is found that the repulsive interactions significantly reduce both the total number of infections and the peak of the pandemic. Consequently, social restrictions allow to flatten the curve. A reduction of the number of infections is also possible by decreasing the amount of motion of the persons.

In particular, we have studied an outbreak after a mass event corresponding to an initial accumulation of persons. The simulations showed that the pandemic develops by radially spreading outwards. In a certain parameter range, an interesting pattern formation effect can be observed, in which the infection spreads in concentric circles that then separate into isolated infection spots. These can be interpreted as infected persons self-isolating at home. As a consequence of demixing, the number of infections is reduced significantly. Further interesting effects were observed when considering a local source of infected persons that corresponds to an airport.

In future work, corresponding DDFT-based models could be constructed relying on compartmental models that are more sophisticated than the standard SIR model, allowing to incorporate, e.g., different age groups or seasonal diseases^11^. In addition, transmission kernels can account for spreading over a certain distance^33^. Moreover, it is possible to perform simulations for outbreaks on larger scales, i.e., in multiple cities or entire countries. Finally, one could investigate the consequences of using different interaction potentials or more sophisticated approximations for the free energy.

## Methods

### Microscopic construction of the model

In this section, we explain how the SIR-DDFT model is constructed from and, consequently, connected to the microscopic dynamics it is a description of. This is of importance for the interpretation of the model, in particular regarding the physical meaning of the transport coefficients. On the microscopic level, persons practicing social distancing can be modeled as particles interacting via repulsive forces^94^. We assume overdamped motion.

It is instructive to consider the limiting case of noninteracting persons first. These will follow independent paths. If we consider motion on local scales, i.e., walking or driving within a city, it is a reasonable approximation to describe it as a standard random walk. When a particle (or person) moves to position **r** in a time *Δt*, the dynamics of the system will be described by a diffusion equation with a diffusion constant^75^

19 $$D={\mathrm{lim}\,}_{\Delta t\to \infty }\frac{\langle \parallel {\bf{r}}(\Delta t)-{\bf{r}}(0){\parallel }^{2}\rangle }{2d\Delta t},$$

where 〈⋅〉 denotes an ensemble average, ∥⋅∥ the Euclidean norm, and *d* ∈ {1, 2, 3} the number of spatial dimensions. Consequently, if *D* is increased, the person moves (on average) a larger distance in a certain time. Mathematically, this is a standard description of a system of noninteracting Brownian particles.

The more general case, which is of particular interest in this work, is that of interacting persons. Staying in the paradigm of Brownian persons, the natural generalization is to assume that the position **r**_*i*_ of person *i* is governed by the Langevin equation^12^

20 $${\dot{{\bf{r}}}}_{i}(t)=\Gamma {{\bf{F}}}_{i}(\{{{\bf{r}}}_{j}\},t)+{{\boldsymbol{\chi }}}_{i}(t),$$

where *Γ* is the person’s mobility, **F**_*i*_ is the force acting on person *i* that depends, in general, on the positions of all persons and on time, and ***χ***_*i*_ is a Gaussian white noise with the properties

21 $$\langle {{\boldsymbol{\chi }}}_{i}(t)\rangle ={\bf{0}},$$

22 $$\langle {{\boldsymbol{\chi }}}_{i}(t)\otimes {{\boldsymbol{\chi }}}_{j}(t^{\prime} )\rangle =2D{\mathbb{1}}{\delta }_{ij}\delta (t-t^{\prime} ).$$

In the latter two equations, $\otimes$ denotes a dyadic product and ${\mathbb{1}}$ the identity matrix of size three. The mobility *Γ* relates the velocity ${\dot{{\bf{r}}}}_{i}$ to the force **F**_*i*_, such that it determines how strongly a person reacts to an applied force. It is related to the diffusion constant by *D* = *Γβ*^−1^ with the rescaled inverse temperature $\beta ={({k}_{B}T)}^{-1}$, where *k*_*B*_ is the Boltzmann constant and *T* the temperature. Consequently, the temperature controls, for a fixed *Γ*, the value of the diffusion constant and thus the amount of random motion. For mixtures with different species of persons, the diffusion constant can have different values for the different species. In our case, a general restriction of social life corresponds to reducing the amount of motion (i.e., the diffusion constant) of all persons, whereas quarantine implies that one specifically isolates infected persons. In the latter case, the diffusion constant *D*_*I*_ of infected persons is reduced. This is a typical way of incorporating quarantine in diffusion models for epidemic spreading^90^.

Although a change of *D*_*I*_ is equivalent to a change of the mobility of infected persons in simple diffusion models, this is not the case in a DDFT for interacting particles, such that we have to choose between changing *Γ*_*I*_ and changing *β*_*I*_ when modeling quarantine. Reducing *Γ*_*I*_ implies, by Eq. (20), that persons exhibit smaller reactions to repulsive forces, which here correspond to social distancing. However, quarantined persons will certainly still try to avoid contacts. Consequently, we have chosen to reduce *D*_*I*_, but not *Γ*_*I*_, which by the relation *D*_*I*_ = *Γ*_*I*_ ${\beta }_{I}^{-1}$ implies that the rescaled temperature ${\beta }_{I}^{-1}$ should be reduced. Although it is, in a real mixture, not easily possible to reduce the temperature of one component while fixing that of the others, it is not problematic in our model where temperature is just a measure for the amount of random motion of the persons. Hence, a reduced amount of motion of infected individuals as a consequence of self-isolation can be implemented by decreasing their temperature. Also reducing *Γ*_*I*_ can be appropriate if the disease itself affects the persons’ ability to move^90^, e.g., by affecting their legs or causing particularly severe symptoms.

As shown by Marini Bettolo Marconi and Tarazona^12^, one can derive from the Langevin equations (20) the DDFT equation (4) governing the ensemble-averaged one-body density *ρ*. The crucial step is the adiabatic approximation, in which it is assumed that the pair correlation of the system equals that of an equilibrium system with the same one-body density. Although this is not exactly true, it has been found to be a good approximation in a large variety of contexts. To get an expression for the free energy (5), we require an approximation for the excess free energy *F*_exc_. We here choose the mean-field approximation

23 $${F}_{{\rm{exc}}}=\frac{1}{2}\mathop{\int}\nolimits_{}^{}{{\rm{d}}}^{d}r\mathop{\int}\nolimits_{}^{}{{\rm{d}}}^{d}r^{\prime} \rho ({\bf{r}},t)\rho ({\bf{r}}^{\prime} ,t){U}_{2}(\parallel {\bf{r}}-{\bf{r}}^{\prime} \parallel ),$$

which provides a good description if the pair-interaction potential *U*_2_ has a Gaussian form^72^.

For mixtures with multiple fields *ρ*_*i*_ corresponding to different particle species *i*, Eq. (4) generalizes to^37^

24 $${\partial }_{t}{\rho }_{i}={\Gamma }_{i}\nabla \cdot \left({\rho }_{i}\nabla \frac{\delta F}{\delta {\rho }_{i}}\right).$$

Here, we consider the specific case of three fields *S*, *I*, and *R* (susceptible, infected, and recovered persons) and assume that they can undergo three types of transitions, which are added to Eq. (24) as nonconserved terms. The first one is infection, which transforms susceptible into infected persons at a rate governed by a parameter *c*. We make the assumption of local transmissions, which can be generalized using transmission kernels^33^. The second one is recovery, which transforms infected into recovered persons at a rate *w*. Finally, constituting the third transition, infected persons can also die at a rate *m*, which removes them from the population. The total density *S* + *I* + *R* is not conserved if we allow for death. We assume (using the language of chemical physics) ideal chemical reactions (see ref. ^39^ for a discussion of the nonideal case).

This model can, in principle, be applied on any scale, both to outbreaks in a city and in an entire country. Simulations on larger scales, however, need to take into account two additional aspects: the first one is the growth of length scales, which is accompanied by a growth of the computational cost. We can increase the computational efficiency by replacing the regions between cities by confining potentials that incorporate the fact that movement from one city to another is less likely than moving within a city.

A second aspect that is relevant on long distances and of crucial importance for disease spreading is air travel. This type of motion differs in two ways from standard Brownian diffusion: first, it allows to travel extremely long distances in a very short time. Second, it is only possible along certain routes and thus depends on the underlying mobility network. The most realistic way of incorporating this aspect is via local sink and source terms that describe airports. A simulation of this type is presented in the fourth part of the results section. The mobility network can be incorporated by coupling the influx at one airport to the outflux at another one. Interestingly, it turns out that the spreading behavior characteristic for diffusion models (of which our theory is a generalization) can also be observed on a global network if the distance between two cities is measured not by spatial distance, but by the effective distance determined by the fraction of traffic between them^89^.

When the underlying mobility network is too complex, an approximate description is useful in terms of Lêvy flights^95^. These can be incorporated into a diffusion equation by using fractional derivatives instead of ordinary derivatives. Models with spatial or temporal fractional derivatives (but without repulsive interactions) have been applied to the spreading of diseases (including COVID-19) before^95,96^. Although a DDFT for particles undergoing Lêvy flights has, to the best of our knowledge, not been derived so far, similar models of the Cahn-Hilliard type^97^ indicate that this should be possible.

### Linear stability analysis

Here, we perform a linear stability analysis of the extended model given by Eqs. (7–9) in one spatial dimension. For the excess free energy, we use a mean-field approximation as in Eqs.  (12–14), but now with general two-body potentials ${U}_{{\rm{sd}}}{h}_{d}(x-x^{\prime} )$ for social distancing and ${U}_{{\rm{si}}}{h}_{i}(x-x^{\prime} )$ for self-isolation. We obtain

25 $${\partial }_{t}S(x,t)	=\, {D}_{S}{\partial }_{x}^{2}S(x,t)-cS(x,t)I(x,t) \\ 	-{\Gamma }_{S}{U}_{{\rm{sd}}}{\partial }_{x}\left(S(x,t){\partial }_{x}\mathop{\int}\nolimits_{}^{}{\rm{d}}x^{\prime} {h}_{d}(x-x^{\prime} ) (S(x^{\prime} ,t)+R(x^{\prime} ,t))\right)\\ 	-{\Gamma }_{S}{U}_{{\rm{si}}}{\partial }_{x}\left(S(x,t){\partial }_{x}\mathop{\int}\nolimits_{}^{}{\rm{d}}x^{\prime} {h}_{i}(x-x^{\prime} )I(x^{\prime} ,t)\right), $$

26 $${\partial }_{t}I(x,t)	=\,{D}_{I}{\partial }_{x}^{2}I(x,t) +cS(x,t)I(x,t)-wI(x,t)\\ 	-{\Gamma }_{I}{U}_{{\rm{si}}}{\partial }_{x}\left(I(x,t){\partial }_{x}\mathop{\int}\nolimits_{}^{}{\rm{d}}x^{\prime} {h}_{i}(x-x^{\prime} )(S(x^{\prime} ,t)+I(x^{\prime} ,t)+R(x^{\prime} ,t))\right),$$

27 $${\partial }_{t}R(x,t)	= \,{D}_{R}{\partial }_{x}^{2}R(x,t)+wI(x,t) \\ 	-{\Gamma }_{R}{U}_{{\rm{sd}}}{\partial }_{x}\left(R(x,t){\partial }_{x}\mathop{\int}\nolimits_{}^{}{\rm{d}}x^{\prime} {h}_{d}(x-x^{\prime} )(S(x^{\prime} ,t)+R(x^{\prime} ,t))\right)\\ 	-{\Gamma }_{R}{U}_{{\rm{si}}}{\partial }_{x}\left(R(x,t){\partial }_{x}\mathop{\int}\nolimits_{}^{}{\rm{d}}x^{\prime} {h}_{i}(x-x^{\prime} )I(x^{\prime} ,t)\right).$$

Any homogeneous state with *S* = *S*_hom_, *R* = *R*_hom_, and *I* = 0, where *S*_hom_ and *R*_hom_ are constants, will be a fixed point. We consider fields $S={S}_{\text{hom}}+\tilde{S}$ and $R={R}_{\text{hom}}+\tilde{R}$ with small perturbations $\tilde{S}$ and $\tilde{R}$ and linearize in the perturbations. This results in

28 $${\partial }_{t}\tilde{S}(x,t)	=\, {D}_{S}{\partial }_{x}^{2}\tilde{S}(x,t)-c{S}_{\text{hom}}I(x,t)\\ 	-{S}_{\text{hom}}{\Gamma }_{S}{U}_{{\rm{sd}}}{\partial }_{x}^{2}\mathop{\int}\nolimits_{}^{}{\rm{d}}x^{\prime} {h}_{d}(x-x^{\prime} )(\tilde{S}(x^{\prime} ,t)+\tilde{R}(x^{\prime} ,t))\\ 	-{S}_{\text{hom}}{\Gamma }_{S}{U}_{{\rm{si}}}{\partial }_{x}^{2}\mathop{\int}\nolimits_{}^{}{\rm{d}}x^{\prime} {h}_{i}(x-x^{\prime} )I(x^{\prime} ,t),$$

29 $${\partial }_{t}I(x,t)=	\,{D}_{I}{\partial }_{x}^{2}I(x,t)+c{S}_{\text{hom}}I(x,t)-wI(x,t),\quad $$

30 $${\partial }_{t}\tilde{R}(x,t)	=\,{D}_{R}{\partial }_{x}^{2}\tilde{R}(x,t)+wI(x,t) \\ 	-{R}_{\text{hom}}{\Gamma }_{R}{U}_{{\rm{sd}}}{\partial }_{x}^{2}\mathop{\int}\nolimits_{}^{}{\rm{d}}x^{\prime} {h}_{d}(x-x^{\prime} )(\tilde{S}(x^{\prime} ,t)+\tilde{R}(x^{\prime} ,t))\hfill\\ 	-{R}_{\text{hom}}{\Gamma }_{R}{U}_{{\rm{si}}}{\partial }_{x}^{2}\mathop{\int}\nolimits_{}^{}{\rm{d}}x^{\prime} {h}_{i}(x-x^{\prime} )I(x^{\prime} ,t).$$

We now drop the tilde and make the ansatz $S={S}_{1}\exp (\lambda t-{\rm{i}}kx)$, $I={I}_{1}\exp (\lambda t-{\rm{i}}kx)$, and $R={R}_{1}\exp (\lambda t-{\rm{i}}kx)$. This gives the eigenvalue equation

31 $$\lambda \left(\begin{array}{l}{S}_{1}\\ {I}_{1}\\ {R}_{1}\end{array}\right)=\left(\begin{array}{lll}-{D}_{S}{k}^{2}+{S}_{\text{hom}}{\Gamma }_{S}{U}_{{\rm{sd}}}{\hat{h}}_{d}(k){k}^{2}&{S}_{\text{hom}}{\Gamma }_{S}{U}_{{\rm{si}}}{\hat{h}}_{i}(k){k}^{2}-c{S}_{\text{hom}}&{S}_{\text{hom}}{\Gamma }_{S}{U}_{{\rm{sd}}}{\hat{h}}_{d}(k){k}^{2}\\ 0&-{D}_{I}{k}^{2}+c{S}_{\text{hom}}-w&0\\ {R}_{\text{hom}}{\Gamma }_{R}{U}_{{\rm{sd}}}{\hat{h}}_{d}(k){k}^{2}&w+{R}_{\text{hom}}{\Gamma }_{R}{U}_{{\rm{si}}}{\hat{h}}_{i}(k){k}^{2}&-{D}_{R}{k}^{2}+{R}_{\text{hom}}{\Gamma }_{R}{U}_{{\rm{sd}}}{\hat{h}}_{d}(k){k}^{2}\end{array}\right)\left(\begin{array}{l}{S}_{1}\\ {I}_{1}\\ {R}_{1}\end{array}\right).$$

Here, ${\hat{h}}_{d}(k)$ and ${\hat{h}}_{i}(k)$ are the Fourier transforms of ${h}_{d}(x-x^{\prime} )$ and ${h}_{i}(x-x^{\prime} )$ (defined as ${\hat{h}}_{j}(k)=\int{\rm{d}}x{h}_{j}(x)\exp ({\rm{i}}kx)$ for *j* = *i*, *d*), respectively. By setting the corresponding characteristic polynomial to zero, we get

32 $$	(-\lambda -{D}_{I}{k}^{2}+c{S}_{\text{hom}}-w)\\ 	\left((-{D}_{S}{k}^{2}+{S}_{\text{hom}}{\Gamma }_{S}{U}_{{\rm{sd}}}{\hat{h}}_{d}(k){k}^{2}-\lambda )\right.\\ 	(-{D}_{R}{k}^{2}+{R}_{\text{hom}}{\Gamma }_{R}{U}_{{\rm{sd}}}{\hat{h}}_{d}(k){k}^{2}-\lambda )\\ 	\left.-{S}_{\text{hom}}{R}_{\text{hom}}{k}^{4}{U}_{{\rm{sd}}}^{2}{\hat{h}}_{d}^{2}(k){\Gamma }_{S}{\Gamma }_{R}\right)=0.$$

The solutions of Eq. (32) are given by the three eigenvalues

33 $${\lambda }_{1}=c{S}_{\text{hom}}-w-{D}_{I}{k}^{2},$$

34 $${\lambda }_{2/3}=	\,\, \frac{{k}^{2}}{2}({U}_{{\rm{sd}}}{\hat{h}}_{d}(k)({S}_{\text{hom}}{\Gamma }_{S}+{R}_{\text{hom}}{\Gamma }_{R})-{D}_{S}-{D}_{R}\\ 	\pm(({U}_{{\rm{sd}}}{\hat{h}}_{d}(k)({S}_{\text{hom}}{\Gamma }_{S}+{R}_{\text{hom}}{\Gamma }_{R})-{D}_{S}-{D}_{R})^{2}\\ 	-4{D}_{S}{D}_{R}+4{U}_{{\rm{sd}}}{\hat{h}}_{d}(k)\left({D}_{S}{R}_{\text{hom}}{\Gamma }_{R} +{D}_{R}{S}_{\text{hom}}{\Gamma }_{S}\right))^{\frac{1}{2}}).$$

A disease outbreak is possible if the real part of *λ*_1_ is positive. This means that the epidemic will start growing when *cS*_hom_ > *w*, as in this case there is a positive eigenvalue. By noting that for a homogeneous distribution, Eq. (18) gives *c*_eff_ = *c*/*A* with the domain area *A*, and that in this case ${\bar{S}}_{0}={S}_{\text{hom}}A$ such that $c{S}_{\text{hom}}={c}_{{\rm{eff}}}{\bar{S}}_{0}$, we can recover the well-known outbreak criterion ${c}_{{\rm{eff}}}{\bar{S}}_{0}\, > \, w$ from the SIR model. When interpreting this result, one should take into account that, as a susceptible person that has been infected cannot become susceptible again, the system will, after a small perturbation, not go back to the same state as before even if *w* > *cS*_hom_. Finally, interaction-related instabilities are accounted for by the eigenvalues *λ*_2_ and *λ*_3_.

### Front speed

For determining the propagation speed of fronts, we can use the marginal stability hypothesis^76–80^. We transform to the co-moving frame that has velocity *v* and assume that the growth rate in this frame is zero at the leading edge. Thereby, we obtain for a general dispersion *λ*(*k*) the equations

35 $${\rm{i}}v+\frac{{\rm{d}}\lambda }{{\rm{d}}k}=0,$$

36 $${\rm{Re}}({\rm{i}}vk+\lambda )=0.$$

These equations can be solved for the complex wavenumber $k={k}_{{\rm{re}}}+{\rm{i}}{k}_{{\rm{im}}}$ and the velocity *v*. For the dispersion *λ*_1_ = *cS*_hom_ − *w* − *D*_*I*_*k*^2^ (we are interested in instabilities associated with infections), Eqs. (35, 36) lead to

37 $${\rm{i}}v-2{\rm{i}}{D}_{I}{k}_{{\rm{im}}}=0,$$

38 $$-2{D}_{I}{k}_{{\rm{re}}}=0,$$

39 $$-v{k}_{{\rm{im}}}+c{S}_{\text{hom}}-w-{D}_{I}({k}_{{\rm{re}}}^{2}-{k}_{{\rm{im}}}^{2})=0.$$

The solution of these equations is

40 $${k}_{{\rm{re}}}=0,$$

41 $${k}_{{\rm{im}}}=\pm \sqrt{\frac{c{S}_{\text{hom}}-w}{{D}_{I}}},$$

42 $$v=2\sqrt{{D}_{I}(c{S}_{\text{hom}}-w)},$$

which is in agreement with results from the literature^28^.

### Basic reproduction number

Here, we discuss how the results from the second part of the results section can be connected to the concept of the basic reproduction number *R*_0_ used in epidemiology, which is defined as the expected number of persons that a single infected individual infects in a completely susceptible population in the absence of control interventions^98^. This number, which is a widely used quantification of disease transmissibility in both scientific and popular literature, has to be introduced and interpreted with care, as it is not a biological property of a disease and often needs to be obtained by sophisticated mathematical modeling. A detailed discussion can be found in ref. ^81^. Some authors do not include the absence of interventions in the definition of *R*_0_^23^. Whether or not this is done affects the results for *R*_0_ obtained in SIR-type models, as will be illustrated below.

If we denote the transmission rate without interventions by *c*_0_, the SIR model gives *R*_0_ = *c*_0_*N*/*w*^98^, where *N* is the population size. Thus, the criterion ${c}_{{\rm{eff}}}{\bar{S}}_{0}\, > \, w$ obtained from a linear stability analysis of the standard SIR model gives

43 $${R}_{0}\frac{{c}_{{\rm{eff}}}}{{c}_{0}}\frac{{\bar{S}}_{0}}{N}\, > \, 1,$$

which, for the case of a completely susceptible population (${\bar{S}}_{0}=N$) and no interventions (*c*_eff_ = *c*_0_), reduces to the well-known criterion *R*_0_ > 1. However, Eq. (43) is more general, as it also holds if the population is not completely susceptible (e.g., if we have a second wave of a pandemic or if a significant fraction of the population is vaccinated) or if there are interventions so that *c*_eff_ ≠ *c*_0_ (e.g., if social distancing measures are implemented). The factor *c*_eff_/*c*_0_ does not appear if *R*_0_ is defined to include interventions. In general, *c*_eff_ is time-dependent and can be reduced below *c*_0_^98^. The fact that the outbreak criterion *R*_0_ > 1 does not hold in general motivates the definition of the (time-dependent) effective reproduction number^98^

44 $${R}_{{\rm{eff}}}=\frac{{c}_{{\rm{eff}}}\bar{S}}{w},$$

which is often simply called *R*^81^ (a convention which we will not adopt in order to avoid confusion with the density of recovered persons). The result of the linear stability analysis then translates into the outbreak criterion *R*_eff_ > 1. In the absence of interventions reducing *c*_eff_ below *c*_0_, we have ${R}_{{\rm{eff}}}={R}_{0}\bar{S}/N$. The same holds if the absence of interventions is not included in the definition of *R*_0_, in which case *R*_0_ = *c*_eff_*N*/*w*.

If we assume that the initial distribution without interventions is a completely susceptible population distributed homogeneously over a domain of area *A*, the instability criterion that follows from the dispersion (17) can be written as

45 $$\frac{c{S}_{\text{hom}}}{w}=\frac{{c}_{0}A{S}_{\text{hom}}}{w}=\frac{{c}_{0}{\bar{S}}_{0}}{w}=\frac{{R}_{0}{\bar{S}}_{0}}{N}\approx {R}_{0}\, > \, 1,$$

where we have used *c* = *c*_0_*A* (from Eq. (18)) in the first, ${\bar{S}}_{0}={S}_{\text{hom}}A$ in the second, *R*_0_ = *c*_0_*N*/*w* in the third, and ${\bar{S}}_{0}\approx N$ in the fourth step. Hence, the outbreak criterion *R*_0_ > 1 still holds in the extended model.

More general insights can be obtained from Eq. (18), which is the microscopic expression for the transmission rate: an inhomogeneous distribution of the population can already be present in the absence of interventions, e.g., owing to the difference in population density between urban and rural areas. This difference is known to affect *R*_0_^81^. We can explain this here by noting that a larger area over which the population is distributed reduces, by Eq. (18), the value of *c*_0_ and thus of *R*_0_.

### Numerical analysis

The simulations for Figs. 1–3 were performed in two spatial dimensions. For Figs. 1 and 2, we used a quadratic domain [0, *L*] × [0, *L*] and for Fig. 3, we chose a circular domain of diameter *L*, where the domain size was set to *L* = 10. To solve the equations of the SIR-DDFT model, we applied an explicit finite-difference scheme with spatial step size d*x* = 0.05 for Fig. 1a, d*x* = 0.0125 for Figs. 1b, c, and 3, and d*x* = 0.02 for Fig. 2 as well as adaptive time steps. As initial conditions for Figs. 1 and 2, we used a Gaussian distribution with amplitude $5/\sqrt{\pi }$ and variance *L*^2^/50 centered at (*x*, *y*) = (*L*/2, *L*/2) for *S*(*x*, *y*, 0) as well as *I*(*x*, *y*, 0) = 0.001*S*(*x*, *y*, 0) and *R*(*x*, *y*, 0) = 0 for the other fields. Thereby, the mean overall density was given by about $\sqrt{\pi }/5\approx 0.35$. For Fig. 3, we used a homogeneous distribution with $S(x,y,0)=\sqrt{\pi }/5$, *I*(*x*, *y*, 0) = 0, and *R*(*x*, *y*, 0) = 0 as initial conditions.

We imposed periodic boundary conditions for Figs. 1 and 2 and Dirichlet boundary conditions for Fig. 3. In the second case, we also added a source term, which is a Gaussian with amplitude *I*_source_ ∈ {0.05, 0.2} and variance *L*^2^/1000, to the right-hand side of Eq. (8). As the effect of the parameters *c* and *w* on the dynamics is known from previous studies of the SIR model, we fixed their values in all simulations to *c* = 1 and *w* = 0.1 to allow for an outbreak. Moreover, we set *Γ*_*S*_  = *Γ*_*I*_ = *Γ*_*R*_ = 1, *D*_*S*_ = *D*_*I*_ = *D*_*R*_ = 0.01, and *σ*_sd_ = *σ*_si_ = 100, with the exception that we allowed *D*_*I*_ to vary (it was given by ${D}_{I}={\it{\Gamma }}_{I}{\beta }_{I}^{-1}$ with fixed *Γ*_*I*_ = 1 and varying *β*_*I*_) for Fig. 2.

The additional one-dimensional simulations presented in Supplementary Note 3 were performed analogously to those for Fig. 1.

## Supplementary information

Supplementary Information

## Data Availability

The source data underlying Figs. 1–3 and Supplementary Figs. 1–6 are provided as Source Data files at 10.5281/zenodo.4034599^99^.

## Code availability

The code used for the numerical solution of the SIR-DDFT model is available at 10.5281/zenodo.4034599^99^.
