# Supplementary material for: Effects of social distancing and isolation on epidemic spreading modeled via dynamical density functional theory
Source: Nat Commun. 2020 Nov 4;11:5576. doi: 10.1038/s41467-020-19024-0 (PMC7643184; doi:10.1038/s41467-020-19024-0)
Supplement: Supplementary file 1 — Supplementary Information [file 41467_2020_19024_MOESM1_ESM.pdf]

Effects of social distancing and isolation on epidemic spreading modeled via dynamical density functional theory

SUPPLEMENTARY INFORMATION

Michael te Vrugt, Jens Bickmann, and Raphael Wittkowski

SUPPLEMENTARY NOTE 1: GENERALIZED MODEL

Here, we present a possible generalization of our SIR-DDFT model. In the main text, we have used the decomposition

$$F_{\mathrm{exc}} = F_{\mathrm{sd}} + F_{\mathrm{si}} \tag{1}$$

with

$$F_{\mathrm{sd}} = -\int \mathrm{d}^d r \int \mathrm{d}^d r'\, C_{\mathrm{sd}} e^{-\sigma_{\mathrm{sd}}(\mathbf{r}-\mathbf{r}')^2} \Big(\frac{1}{2}S(\mathbf{r},t)S(\mathbf{r}',t) + S(\mathbf{r},t)R(\mathbf{r}',t) + \frac{1}{2}R(\mathbf{r},t)R(\mathbf{r}',t)\Big), \tag{2}$$

$$F_{\mathrm{si}} = -\int \mathrm{d}^d r \int \mathrm{d}^d r'\, C_{\mathrm{si}} e^{-\sigma_{\mathrm{si}}(\mathbf{r}-\mathbf{r}')^2} I(\mathbf{r},t)\Big(\frac{1}{2}I(\mathbf{r}',t) + S(\mathbf{r}',t) + R(\mathbf{r}',t)\Big), \tag{3}$$

which gives the excess free energy (i.e., the contribution from interactions) as a sum of social distancing and self-isolation. Instead, one can use the form

$$F_{\mathrm{exc}} = F_{\mathrm{sd}} + F_{\mathrm{iso}} + F_{\mathrm{ill}}. \tag{4}$$

In this case, social distancing remains unaffected. However, there are now two terms F_{iso} and F_{ill} determining the way infected persons interact with others. F_{iso} is the isolation term, which corresponds to a repulsive interaction between infected and healthy individuals. The term F_{ill} models the interaction of infected persons with other infected persons. This can have various forms. They repel each other if they practice social distancing or self-isolation, but they can also attract each other (e.g., if they intentionally accumulate in a hospital or quarantine station). Assuming that the interaction corresponding to F_{ill} is also Gaussian, i.e.,

$$F_{\mathrm{iso}} = -\int \mathrm{d}^d r \int \mathrm{d}^d r'\, C_{\mathrm{iso}} e^{-\sigma_{\mathrm{iso}}(\mathbf{r}-\mathbf{r}')^2} I(\mathbf{r},t)(S(\mathbf{r}',t) + R(\mathbf{r}',t)), \tag{5}$$

$$F_{\mathrm{ill}} = -\frac{1}{2}\int \mathrm{d}^d r \int \mathrm{d}^d r'\, C_{\mathrm{ill}} e^{-\sigma_{\mathrm{ill}}(\mathbf{r}-\mathbf{r}')^2} I(\mathbf{r},t)I(\mathbf{r}',t) \tag{6}$$

with the parameters C_{iso} and C_{ill} for the strength and σ_{iso} and σ_{ill} for the range of the infected-noninfected and infected-infected interactions, respectively, the model given by Equations (12)-(14) in the main text generalizes to

$$\partial_t S = D_S \nabla^2 S - \Gamma_S \nabla \cdot \big(S\nabla(C_{\mathrm{sd}}K_{\mathrm{sd}} \star (S+R) + C_{\mathrm{iso}}K_{\mathrm{iso}} \star I)\big) - cSI, \tag{7}$$

$$\partial_t I = D_I \nabla^2 I - \Gamma_I \nabla \cdot \big(I\nabla(C_{\mathrm{iso}}K_{\mathrm{iso}} \star (S+R) + C_{\mathrm{ill}}K_{\mathrm{ill}} \star I)\big) + cSI - wI, \tag{8}$$

$$\partial_t R = D_R \nabla^2 R - \Gamma_R \nabla \cdot \big(R\nabla(C_{\mathrm{sd}}K_{\mathrm{sd}} \star (S+R) + C_{\mathrm{iso}}K_{\mathrm{iso}} \star I)\big) + wI \tag{9}$$

with the kernels

$$K_{\mathrm{iso}}(\mathbf{r}) = e^{-\sigma_{\mathrm{iso}}\mathbf{r}^2}, \tag{10}$$

$$K_{\mathrm{ill}}(\mathbf{r}) = e^{-\sigma_{\mathrm{ill}}\mathbf{r}^2} \tag{11}$$

and K_{sd} as defined in the main text. For $C_{\mathrm{iso}} = C_{\mathrm{ill}} = C_{\mathrm{si}}$ and $\sigma_{\mathrm{iso}} = \sigma_{\mathrm{ill}} = \sigma_{\mathrm{si}}$, the standard case is recovered. The general model can also allow for attractive interactions between infected persons, or simply for a reduction of the repulsion between them (resulting from the fact that they are already ill).

SUPPLEMENTARY NOTE 2: EFFECTIVE TRANSMISSION RATE

As discussed in the main text, many key features of the dynamics in the SIR-DDFT model can be understood by mapping it onto a standard SIR model using the effective transmission rate c_{eff}. It is defined by the requirement that the rate of change of the total number of susceptible ($\bar{S}$) and infected ($\bar{I}$) persons satisfies (for death rate $m = 0$)

$$\dot{\bar{S}} = -c_{\mathrm{eff}}\bar{S}\bar{I}, \tag{12}$$

$$\dot{\bar{I}} = c_{\mathrm{eff}}\bar{S}\bar{I} - w\bar{I}. \tag{13}$$

Integrating the SIR-DDFT model over space shows that such a parameter c_{eff} exists and is given by

$$c_{\mathrm{eff}}(t) = c\int \mathrm{d}^d r\, e_S(\mathbf{r},t)e_I(\mathbf{r},t) \tag{14}$$

with the normalized distributions $e_S = S/\bar{S}$ and $e_I = I/\bar{I}$. When data for the time evolution of $\bar{S}$ and $\bar{I}$ are given, Supplementary Equations (12) and (13) allow to obtain the effective transmission rate as

$$c_{\mathrm{eff}} = -\frac{\dot{\bar{S}}}{\bar{S}\bar{I}} \tag{15}$$

or

$$c_{\mathrm{eff}} = \frac{\dot{\bar{I}} + w\bar{I}}{\bar{S}\bar{I}}. \tag{16}$$

We have used Supplementary Equation (16) to obtain c_{eff} as a function of time for the three time evolutions shown in Figure 1(b) of the main text, which correspond to the parameter values $C_{\mathrm{si}} = C_{\mathrm{sd}} = 0$ (no interactions), $C_{\mathrm{si}} = 2C_{\mathrm{sd}} = -20$ (moderate interactions), and $C_{\mathrm{si}} = 3C_{\mathrm{sd}} = -30$ (strong interactions). For better visualization, the curves have been smoothened using a Savitzky-Golay filter. Since Figure 1(b) in the main text presents the normalized values $\bar{S}_{\mathrm{n}}$, $\bar{I}_{\mathrm{n}}$, and $\bar{R}_{\mathrm{n}}$, we here give the rescaled effective transmission rate $c_{\mathrm{eff,n}} = Nc_{\mathrm{eff}}$ with the population size N that one has to use if Supplementary Equations (12) and (13) are written for $\bar{S}_{\mathrm{n}} = \bar{S}/N$ and $\bar{I}_{\mathrm{n}} = \bar{I}/N$ rather than $\bar{S}$ and $\bar{I}$. We focus on the time interval $[0, 50]$, since the main infection dynamics takes place during this period. The results are shown in Supplementary Figure 1. At the beginning, the values of $c_{\mathrm{eff,n}}$ are the same in all simulations, which is a consequence of the identical initial conditions. In the noninteracting case, one observes an increase of $c_{\mathrm{eff,n}}$ to a value of about 2.21 at time $t \approx 2$, followed by a strong decrease. For the simulations with interactions, it is found that $c_{\mathrm{eff,n}}$ decreases at the beginning. In the case of strong interactions, a sharp reduction of $c_{\mathrm{eff,n}}$ from about 0.62 to about 0.17 is observed between times $t = 6$ and $t = 8$. After the initial decay, a regime with a relatively constant value of $c_{\mathrm{eff,n}}$ is reached in all three simulations. The value at $t = 50$ is given by $c_{\mathrm{eff,n}} \approx 0.44$ for $C_{\mathrm{si}} = 2C_{\mathrm{sd}} = -20$, $c_{\mathrm{eff,n}} \approx 0.29$ for $C_{\mathrm{si}} = C_{\mathrm{sd}} = 0$, and $c_{\mathrm{eff,n}} \approx 0.24$ for $C_{\mathrm{si}} = 3C_{\mathrm{sd}} = -30$.

For the noninteracting case, the values of $c_{\mathrm{eff,n}}$ in the initial spreading phase are relatively large, such that the number of infected persons can grow rapidly. As indicated in the main text, the time evolution of $c_{\mathrm{eff,n}}$ for moderate interactions also explains the results for $\bar{I}_{\mathrm{max,n}}$ and $\bar{S}_{\infty,\mathrm{n}}$ from the main text: In the presence of interactions, the transmission rate is lower during the initial spreading phase, leading to a reduction of the peak value $\bar{I}_{\mathrm{max,n}}$. On the other hand, the final transmission rate is higher for moderate than for no interactions, such that $\bar{S}_{\infty,\mathrm{n}}$ is slightly lower in the former case. Finally, the sharp decrease of $c_{\mathrm{eff,n}}$ in the case of strong interactions can be explained by the onset of pattern formation, which greatly reduces the spatial overlap of S and I and therefore, by Supplementary Equation (14), the value of the effective transmission rate.

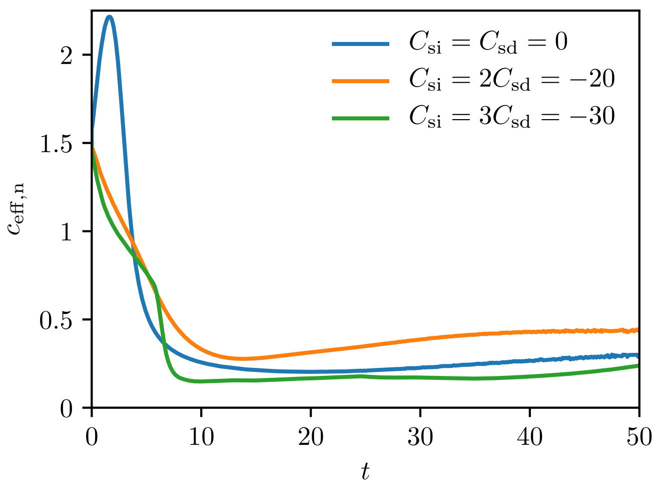

Supplementary Figure 1. Time evolution of the rescaled effective transmission rate $c_{\mathrm{eff,n}}$ for the simulations presented in Figure 1 of the main text. Initially, large transmission rates are observed for the case of no interactions ($C_{\mathrm{si}} = C_{\mathrm{sd}} = 0$), explaining the corresponding large value of $\bar{I}_{\mathrm{max,n}}$. At later times, the value of $c_{\mathrm{eff,n}}$ is larger for moderate interactions ($C_{\mathrm{si}} = 2C_{\mathrm{sd}} = -20$). The smallest value of $c_{\mathrm{eff,n}}$ is reached for strong interactions ($C_{\mathrm{si}} = 3C_{\mathrm{sd}} = -30$) that lead to phase separation.

SUPPLEMENTARY NOTE 3: SIMULATIONS IN ONE SPATIAL DIMENSION

In the main text, we have presented simulation results for an outbreak in a two-dimensional (2D) system. However, it is also of interest how the model behaves in one spatial dimension (1D). Analytical investigations and numerical simulations are easier to perform in 1D, whereas the 2D case is more realistic given that humans live on a two-dimensional surface. A second parameter that is of potential relevance and that will also be considered here is the domain size.

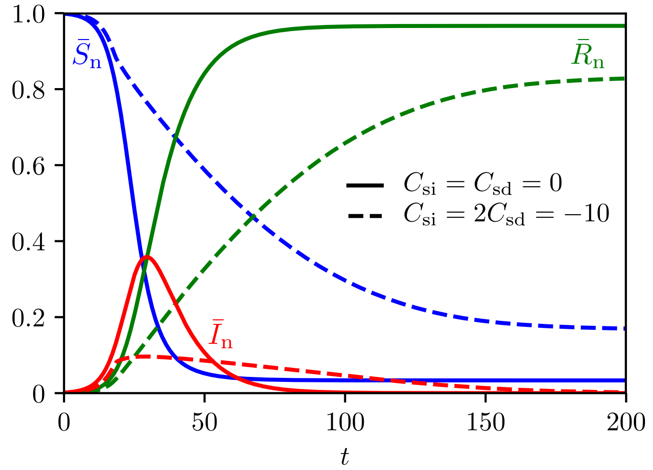

Supplementary Figure 2. Time evolution of the fraction of susceptible, infected, and recovered persons for domain size $L = 1$. The time evolution is shown for no interactions ($C_{\mathrm{si}} = C_{\mathrm{sd}} = 0$) and for interactions with $C_{\mathrm{si}} = 2C_{\mathrm{sd}} = -10$. The curve $\bar{I}_{\mathrm{n}}$ is flatter and has a smaller peak in the presence of repulsive interactions as is the case in 2D.

To investigate these aspects, we have numerically solved the model in one spatial dimension for domain sizes $L = 1$ and $L = 10$. The spatial step size used was $\mathrm{d}x = 0.005$ (individual simulations) or $\mathrm{d}x = 0.01$ (parameter scan). As an initial condition, we used a Gaussian with amplitude 1 and variance $L^2/50$. If not specified otherwise in the figures or corresponding captions, all parameters have the values used for Figure 1 of the main text (see the discussion of the numerical methods). The results for $L = 1$ are shown in Supplementary Figure 2, presenting the fraction of susceptible, infected, and recovered persons as a function of time t, and in Supplementary Figure 3, visualizing the spatiotemporal evolution of $I(x,t)$ as a function of position x and time. As is obvious, the main effects – inhibition of the outbreak by repulsive interactions and formation of infection clusters – are also present in 1D. (Since there is no difference between rings and spots in 1D, we refer to the regions of high infection concentration as 'infection clusters'.) An advantage of working in 1D is that a visualization of the complete time evolution of $I(x,t)$ in the form of a spacetime plot (Supplementary Figure 3) is possible.

In Supplementary Figures 4 and 5, the same plots are shown for a system with $L = 10$. By comparing Supplementary Figures 2 and 4, showing the fractions of persons in the various compartments, it is found that the general result – repulsive interactions lead to a reduction of the outbreak – is not affected by the domain size. A stronger difference is observed when comparing Supplementary Figures 3 and 5: The number of infection clusters is much larger in the case of a larger domain. Moreover, infection clusters occasionally merge. The general picture (infected persons self-organize into small clusters) is in agreement with the 2D result. In particular, the fact that increasing the domain size leads to a large number of small clusters rather than to a small number of large clusters supports our interpretation of the phase separation as infected persons self-isolating at home proposed in the main text. The formation of states with multiple spikes and transitions between them in an infection model has also been discussed in Ref. [1], where phase separation was interpreted as quarantine.

Finally, Supplementary Figure 6 shows the phase diagrams of the SIR-DDFT model in 1D for a domain size $L = 1$. As in the 2D case, a phase transition associated with a reduction of the outbreak and with demixing is observed in the top left corner of the phase diagrams. A difference is the location of the phase boundary: In 2D, larger values of the interaction parameters are required for phase separation. This observation is plausible when comparing it to results for the demixing of GCM fluids by Louis et al. [2], who found that the location of the phase boundary depends on the zero-wavelength value of the Fourier-transformed Gaussian interaction potential. This is proportional to the range of the repulsive interaction in 1D and to its square in 2D, such that – for equal parameter values – the quantitative results change. The region in the bottom left corner of the phase diagrams for the large-domain simulation in 2D, in

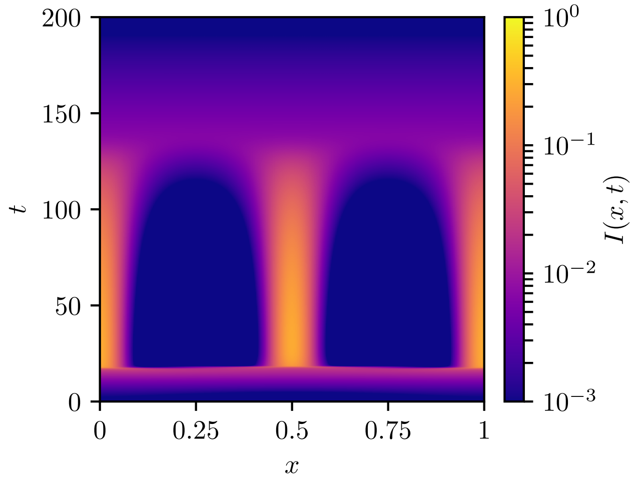

Supplementary Figure 3. Density of infected persons $I(x,t)$ as a function of position x and time t for domain size $L = 1$ and interaction strength $C_{si} = 2C_{sd} = -10$. The formation of infection clusters is also observed in 1D.

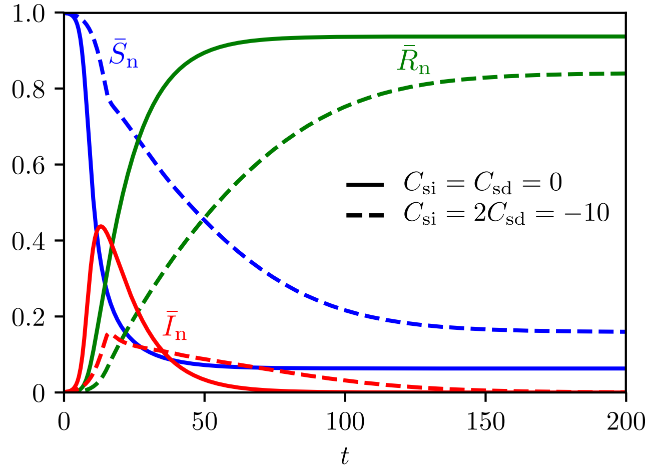

Supplementary Figure 4. The same as in Supplementary Figure 2, but now for domain size $L = 10$. For a larger domain size, repulsive interactions still lead to a lower number of infections.

which diffusive spreading is more important than phase separation, is not visible in Supplementary Figure 6 (demixing is the dominant effect).

In summary, it was found that, while certain quantitative differences arise, most of the main qualitative results are not affected by changing the number of spatial dimensions or the domain size. This is an important advantage for the practical applicability of the model, as it implies that useful insights can already be gained from solving it in 1D on small domains, which greatly reduces the computational cost. Important exceptions include the difference between infection rings and spots and the existence of a region with reduced spreading in the bottom left corner of the phase diagram.

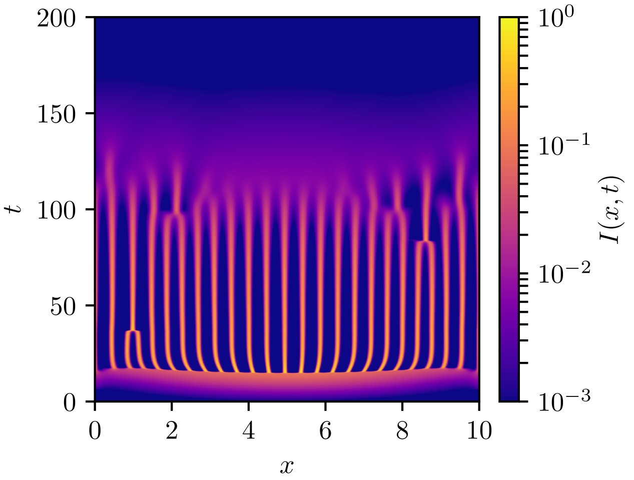

Supplementary Figure 5. The same as in Supplementary Figure 3, but now for domain size $L = 10$. Enlarging the domain leads to an increase of the number of infection clusters and a more complex time evolution.

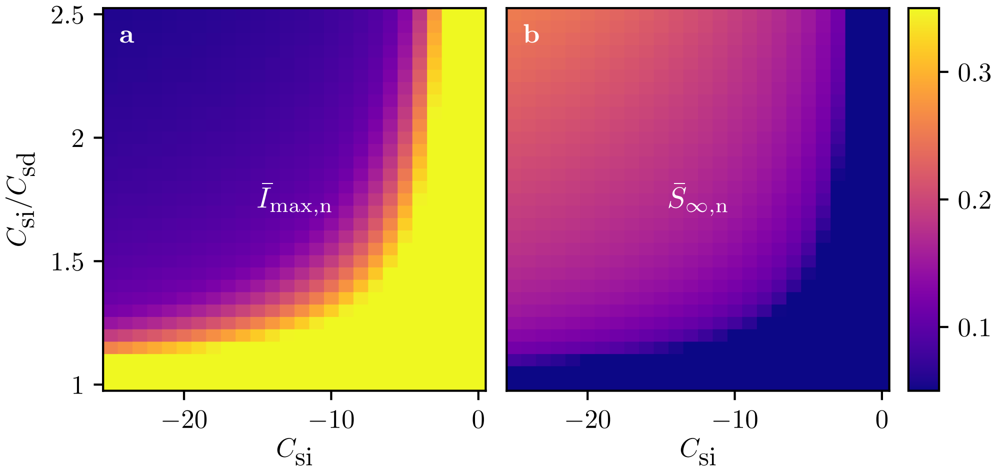

Supplementary Figure 6. Phase diagrams for the SIR-DDFT model in 1D for domain size $L = 1$. The dependence of the maximal fraction of infected persons $\bar{I}_{\mathrm{max,n}}$ and the final fraction of susceptible persons $\bar{S}_{\infty,\mathrm{n}}$ on the strength of self-isolation C_{si} and social distancing C_{sd} is shown. The phase boundary arises in the same way as in 2D, although at different parameter values.

SUPPLEMENTARY REFERENCES

[1] Gai, C., Iron, D. & Kolokolnikov, T. Localized outbreaks in an SIR model with diffusion. *J. Math. Biol.* **80**, 1389–1411 (2020).

[2] Louis, A. A., Bolhuis, P. G. & Hansen, J. P. Mean-field fluid behavior of the Gaussian core model. *Phys. Rev. E* **62**, 7961–7972 (2000).
